# The Role of Gold Nanorods in the Response of Prostate Cancer and Normal Prostate Cells to Ionizing Radiation—In Vitro Model

**DOI:** 10.3390/ijms22010016

**Published:** 2020-12-22

**Authors:** Marika Musielak, Agnieszka Boś-Liedke, Igor Piotrowski, Maciej Kozak, Wiktoria Suchorska

**Affiliations:** 1 Radiobiology Laboratory, Department of Medical Physics, Greater Poland Cancer Centre, 61-866 Poznań, Poland; igor.piotrowski@wco.pl (I.P.); wiktoria.suchorska@wco.pl (W.S.); 2 Department of Macromolecular Physics, Faculty of Physics, Adam Mickiewicz University, 61-614 Poznań, Poland; agnbos@amu.edu.pl (A.B.-L.); mkozak@amu.edu.pl (M.K.); 3 Department of Electroradiology, Poznan University of Medical Sciences, 61-701 Poznań, Poland

**Keywords:** gold nanorods, nanoparticles, radiotherapy, prostate cancer, radiosensitivity

## Abstract

To increase the efficiency of therapy via enhancing its selectivity, the usage of gold nanorods (GNR) as a factor sensitizing cancer cells to radiation was proposed. Due to gold nanoparticles’ characteristics, the smaller doses of radiation would be sufficient in the treatment, protecting the healthy tissue around the tumor. The aim of this study was to investigate the effect of gold nanorods on cancer and normal prostate cells and the role of nanorods in the cell response to ionizing radiation. The effect was evaluated by measuring the toxicity, cell cycle, cell granularity, reactive oxygen species (ROS) level, and survival fractions. Nanorods showed a strong toxicity dependent on the concentration and incubation time toward all used cell lines. A slight effect of nanorods on the cycle distribution was observed. The results demonstrated that the administration of nanorods at higher concentrations resulted in an increased level of generated radicals. The results of cellular proliferation after irradiation are ambiguous; however, there are noticeable differences after the application of nanorods before irradiation. The obtained results lead to the conclusion that nanorods affect the physiology of both normal and cancer cells. Nanorods might become a potential tool used to increase the effectiveness of radiation treatment

## 1. Introduction

Prostate cancer is the most common cancer among men in developed countries, and at the same time, its etiology is unclear [1]. It has one of the highest rates of incidence and prevalence among all malignancies, second only to the lung cancer as the most common cancer diagnosed in men around the world. It is also one of the main causes of deaths due to cancer in men [1,2]. 

During the prostate cancer treatment, the radiation therapy is used as an alternative to the radical surgery. There are two main forms of radiotherapy, i.e., external radiotherapy and brachytherapy. In order to minimize the risk of complications while improving the results of treatment, the standard procedure includes the use of ionizing radiation doses above 70 Gy and conformal techniques such as image guided radiotherapy (IGRT) and radiation modulation based on intensity modulated radiation therapy (IMRT). Prostate cancer is characterized by relatively low sensitivity to radiation [3]. Due to the non-selective activity of ionizing radiation, effects on both cancer and normal cells are observed. In order to mitigate the harmful side effects by minimizing the field of exposure, a new, improved therapeutic approach that allows for better specificity against cancer cells is needed [4]. Such a method will have to show increased cytotoxicity toward cancer cells and reduced side effects. In the light of the disadvantages of current treatment methods, one of the most promising resolutions can be found in nanotechnology research. It includes structures with at least one of the dimensions less than or equal to 100 nm. The most important feature of materials at the nanometric scale is the change in their physicochemical properties along with the change in size. Consequently, nanometric materials differ significantly from their counterparts at the macro scale. 

Gold nanoparticles (GNP) are characterized by a high ratio of active surface to volume, the ability to modify their surface, and optical properties associated with the occurrence of surface plasmons. A potential strategy to solve the problem of the protection of normal tissues in the beam field could be the introduction of gold nanoparticles into the tumor area. The change in radiation absorption properties would allow the use of lower doses of radiation, which would lower the negative effects on the normal tissue in the vicinity of the tumor. In order to induce an increased cellular response to ionizing radiation, a high atomic number (Z) material, such as gold, should be used [5]. The enhanced therapeutic effect, while using keV radiation, is caused by the occurrence of the photoelectric effect. Due to the high atomic number of gold, the probability of generating a photoelectron cascade and a larger number of Auger electrons increases. Much attention was devoted to the research on the dependence of radiation sensitivity at clinically relevant energies. Chithrani et al. [6] observed a greater potential of radiation sensitization for cells irradiated with lower energy beams for photon sources. There remains uncertainty if gold nanoparticles cause a cytotoxic response. Some authors attribute cytotoxicity to particular preparation protocols or nanoparticle sizes, while others find that gold nanoparticles do not cause any immunogenic or cytotoxic effects [7,8]. Another work [9] examined glucose-coated gold nanoparticles (Glu-GNPs), which were used to improve cellular targeting and radiation sensitivity. In this study, a mechanism of increased radiation sensitivity was observed following the incubation of radiation-resistant human prostate cancer cells with Glu-GNPs. The authors demonstrated that treatment with Glu-GNP followed by irradiation with 2 Gy dose resulted in a stronger inhibition of proliferation compared with irradiation alone. Glu-GNPs induced acceleration in the G0/G1 phase and accumulation of cells in the G2/M phase compared to the control. Glu-GNPs also triggered the activation of cyclin-dependent kinases leading to acceleration of the cell cycle and, most importantly, accumulation in the G2/M phase. This activation was accompanied by a sensitizing effect on ionizing radiation, which may have clinical implications. 

The aim of our study was to analyze the effect of elongated gold nanoparticles—gold nanorods (GNR)—as a factor changing the efficiency of ionizing radiation on a model system—human prostate cancer cell lines LNCaP and PC-3 and human normal prostate cells PNT1A in vitro. These cell lines were chosen because of different proliferative and hormonal profiles. The specific aims of the research were (1) the assessment of toxicity of gold nanoparticles and analysis of the maximum uptake of gold nanoparticles by the cells, (2) the analysis of the impact of gold nanoparticles on cell cycle distribution, (3) the analysis of the impact of gold nanoparticles on the quantitative production of reactive oxygen species (ROS), (4) the analysis of the impact of gold nanoparticles on enhancing the sensitivity to ionizing radiation, and (5) the evaluation of cell proliferation as a parameter characterizing the survival fraction after applied toxic factors.

## 2. Results

### 2.1. Nanorods Morphology (TEM)

**Figure 1:**
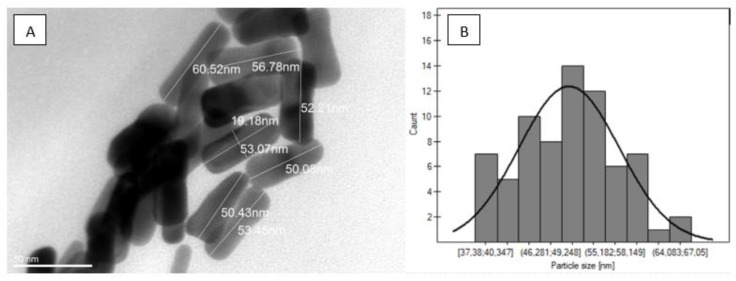
Nanorods morphology. (**A**): TEM images of gold nanorods used in the study. (**B**): An average size distribution of gold nanorods.

The morphology of synthesized gold nanorods was assessed by transmission electron microscopy. The average length of obtained nanorods reached about 50 nm (+/−6 nm) (Figure 1). 

### 2.2. Toxicity

**Figure 2:**
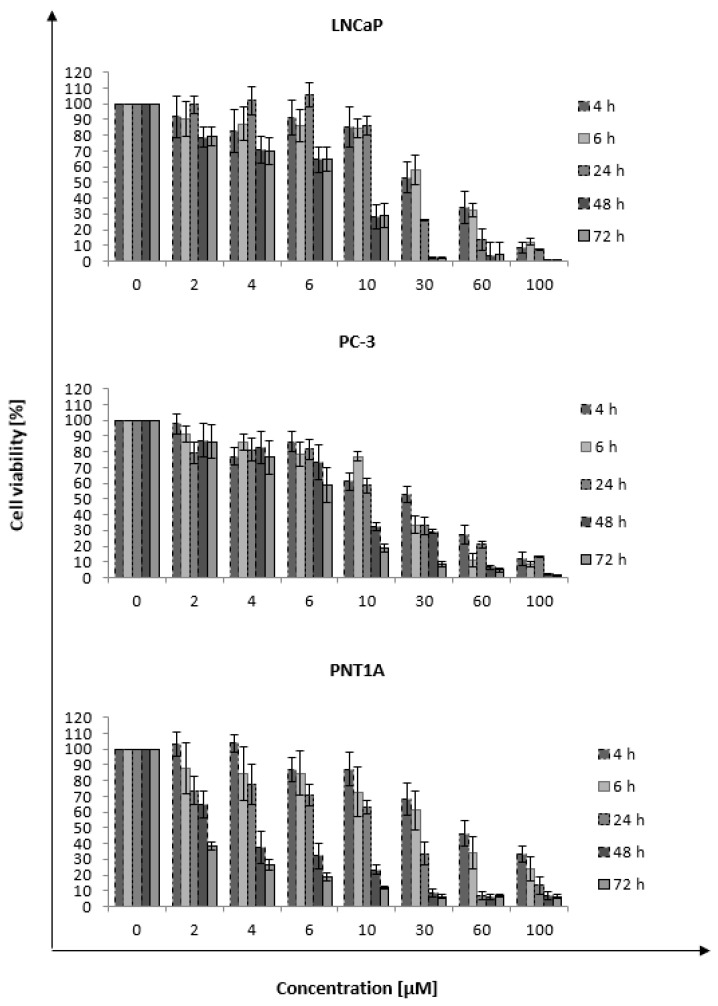
The effect of incubation with gold nanorods on the viability of LNCaP, PC-3, and PNT1A cells. The values presented in the graph are the mean of the obtained results and the deviation is the standard deviation. The experiment was performed in triplicate.

The first test to assess the impact of nanorods on cancer and normal cells was the MTT assay determining the GNR toxicity. This test measures the metabolic activity of the LNCaP, PC-3, and PNT1A cells expressed as a percentage of cell viability (untreated cells set as 100%). The gold nanorods at concentrations: 2, 4, 6, 10, 30, 60, and 100 μM were used, and their impact on the cell viability at five time points—4, 6, 24, 48, 72 h was measured (Figure 2). A strong dependence of GNR concentration on the viability of LNCaP, PC-3, and PNT1A cells was observed. The level of metabolic activity was dependent on the concentration of nanorods, and the cell viability decreased with the increasing concentration of GNRs. Moreover, increased incubation time with nanorods also resulted in lower cell viability. The highest cytotoxicity was observed for each cell line after 72 h of incubation. Comparing the highest and lowest IC50 values after 4 h of incubation with GNR, the normal cells were the most resistant to nanorods, while the most sensitive was LNCaP. Some deviation was also noticeable, since the 24-h incubation time resulted in lower cytotoxicity in viability than the 6-h incubation time in the concentration range up to 30 μM The PNT1A line showed a similar response to GNR during the 48 and 72 h incubation.

### 2.3. Nanorods Interaction

**Figure 3:**
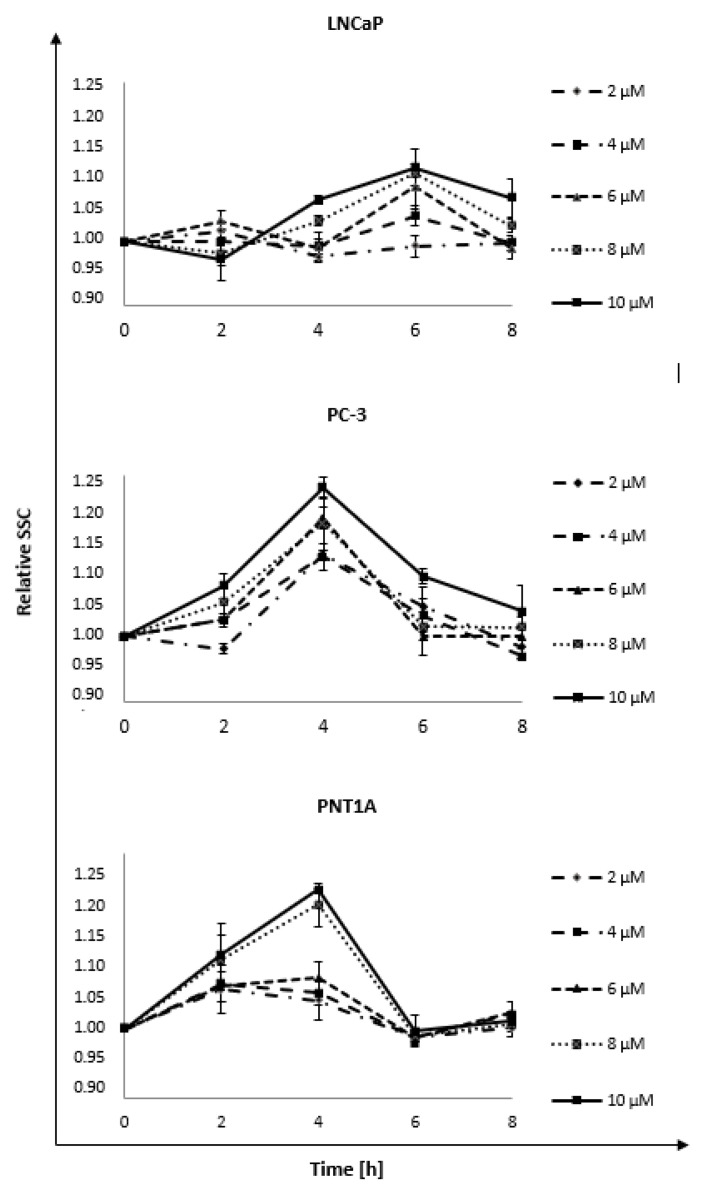
The dynamic of gold nanorods uptake by LNCaP, PC-3, and PNT1A cells. The analysis was performed at four time points 2, 4, 6, and 8 h for five concentrations of nanorods: 2, 4, 6, 8, and 10 μM. The values presented in the graph are the mean of the obtained results, and the deviation is the standard deviation. The experiment was performed in triplicate.

The nanorods interaction (uptake and surface adsorption) with LNCaP, PC-3, and PNT1A cell lines was determined based on the cytometric analysis of the SSC parameter reflecting cellular granularity. The measurement was made with the assumption that the more nanorods a cell absorbs, the more granular it will be. For this purpose, analysis was performed at four time points—2, 4, 6, and 8 h—for five concentrations of nanorods: 2, 4, 6, 8, and 10 μM (Figure 3). Those concentrations were chosen because of minimal cytotoxic effects on PNT1A cells. The obtained results showed concentration-dependent changes in relative granularity, indicating the internalization and adsorption of nanorods in both normal and cancer cells. We observed a noticeable increase in granularity up to 4 h (PC-3 and PNT1A) and 6 h (LNCaP), which was followed by its decrease. Presumably, this indicates the removal of nanorods from the inside of the cells after prolonged incubation. The LNCaP line showed the highest maximum granularity at 6 h, which was followed by a less pronounced decrease than that observed in two other cell lines. For LNCaP, the normalized SSC increased by 0.11 ± 0.02 at 6 h for the highest concentration. In the case of the PC-3 line, the highest granularity was observed at a 4-h time point, with an increase of 0.22 ± 0.02 compared to control. Increasing values in granularity could be observed after using higher concentrations of nanorods. The PNT1A line had a very similar dynamic interaction as the PC-3 line. Maximum SSC occurred after 4 h, the increase was 0.19 ± 0.01 compared to control, while in 6 h, it decreased drastically. The increase in granularity was observed after incubation with 8 μM and 10 μM GNRs.

### 2.4. Cell Cycle Distribution

**Figure 4:**
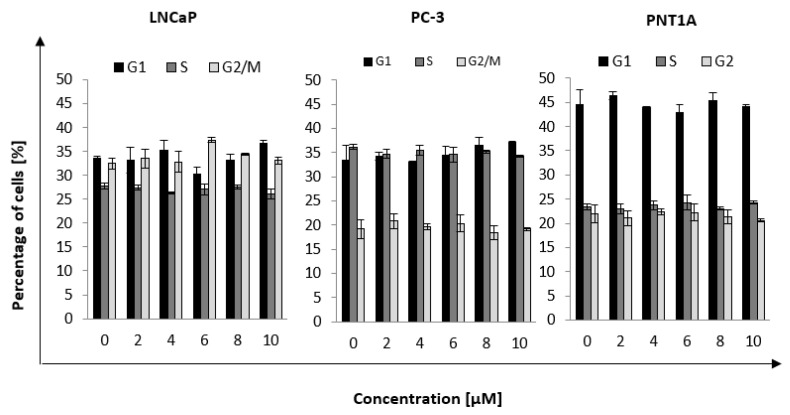
Percentage of cells in the G1, S, and G2 phases of cell cycle, depending on the used nanorods concentration. The values presented in the graph are the mean of the obtained results and, the deviation is the standard deviation. The experiment was performed in triplicate.

The next step was to determine the percentage of cells in a given phase of the cell cycle after incubation with nanorods for times at which maximum uptake was observed. The selected time points were: LNCaP, 6 h; PC, 4 h; and PNT1A, 4 h (Figure 4). Five concentrations of nanorods were selected based on toxicity results. Less toxic concentrations were chosen, as the goal was to measure the enhancement of the radiation effect and not the toxicity of the nanorods. We observed an increase in the number of LNCaP cells in the G2 phase of 5% ± 0.9% for a concentration of 6 μM compared to the control. In the case of the PC-3 and PNT1A cell lines, no discernible differences in the cell cycle distribution could be observed. 

### 2.5. ROS Production

**Figure 5:**
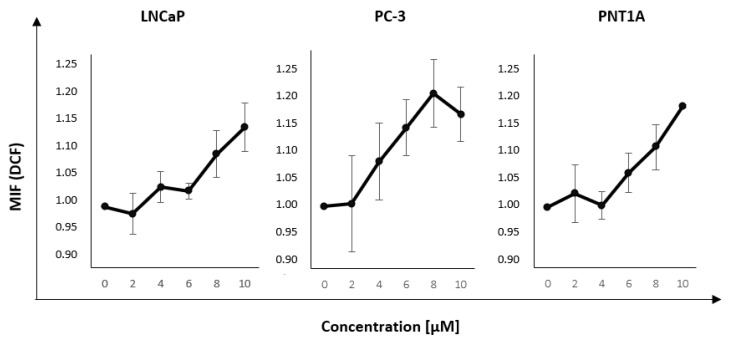
Results of reactive oxygen species in cells after incubation with nanorods. Mean fluorescence intensity (MIF) parameter determined the number of reactive oxygen species (ROS) generated depending on the nanorods concentration in the LNCaP cell line after 6 h incubation, PC-3 cell line after 4 h incubation, and PNT1A cell line after 4 h incubation. The values presented in the graph are the mean of the obtained results, and the deviation is the standard deviation. The experiment was performed in triplicate.

Using the results determining the toxicity of nanorods and the time point at which the uptake is the highest (LNCaP—6 h, PC-3—4 h, PNT1A—4 h) after incubation with GNR, the measurement of ROS level was performed. The aim was to measure the level of reactive oxygen species after incubation with GNR in the concentration of range of 2–10 μM. The results are presented in Figure 5. An increase in the mean fluorescence intensity indicated a higher level of reactive oxygen species formed. The obtained results showed an increased level of reactive oxygen species after incubation with nanorods; however, the changes were not statistically significant. We observed that incubation with GNR at higher concentrations resulted in higher ROS level in all three cell lines.

### 2.6. Surviving Fraction after Incubation with GNRs Followed by Irradiation 

**Figure 6:**
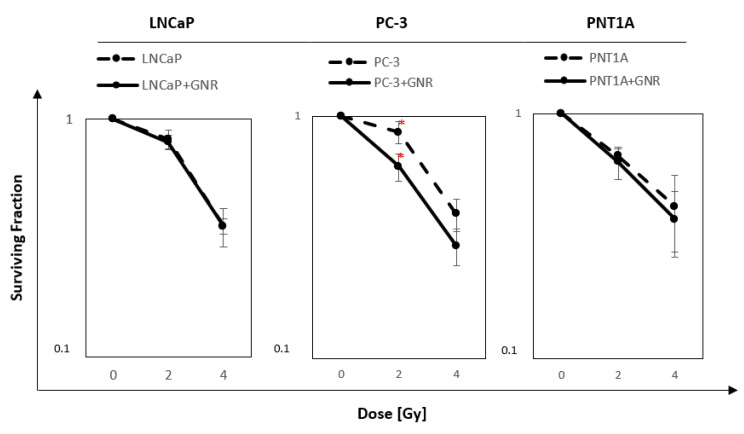
Survival curves after irradiation with 2 and 4 Gy doses of cells previously incubated with 6 μM GNR for a time determined by the highest value of nanorods absorption by selected cell lines. Survival curves for LNCaP after 6 h of incubation time, for PC-3 after 4 h of incubation time, and for PNT1A after 4 h of incubation time. Values with red “*” mean the only statistically significant difference (*p* < 0.05) between values in the point of 2 Gy. The values presented in the graph are the mean of the obtained results, and the deviation is the standard deviation. The experiment was performed in triplicate.

Next, we decided to measure the enhancing effect of GNRs on radiation response. For this analysis, the cells were incubated with 6 μM GNRs for the time ensuring the highest uptake, and then irradiated. Both prostate cancer and normal cells were treated with 2 and 4 Gy doses. LNCaP cells were irradiated after 6 h incubation, and PC-3 and PNT1A cells were irradiated after 4 h of incubation with 6 μM GNR. The results of the clonogenic tests are presented in Figure 6. In the LNCaP cells, no difference was observed between cells irradiated after incubation with nanorods and cells that underwent irradiation alone (*p* = 0.71 for 2 Gy; *p* = 0.98 for 4 Gy). The different relationship was observed for the PC-3 cells. A statistically significant difference was noted between the SF of cells treated with 2 Gy dose alone and cells treated with GNR and 2 Gy dose (*p* = 0.04). The difference in the surviving fraction between the two groups was 0.24 in that point. In the cells exposed to 4 Gy, no difference was observed between cells irradiated following incubation with GNRs and the cells irradiated without GNR (*p* = 0.49). PNT1A cells showed no statistically significant difference at 2 Gy, comparing cells incubated with and without GNRs (*p* = 0.71). Analyzing cells exposed to 4 Gy with nanorods and cells treated with 4 Gy alone, no difference (*p* = 0.72) could be seen.

### 2.7. Cell Cycle Distribution after Incubation with GNR and Irradiation 

**Figure 7:**
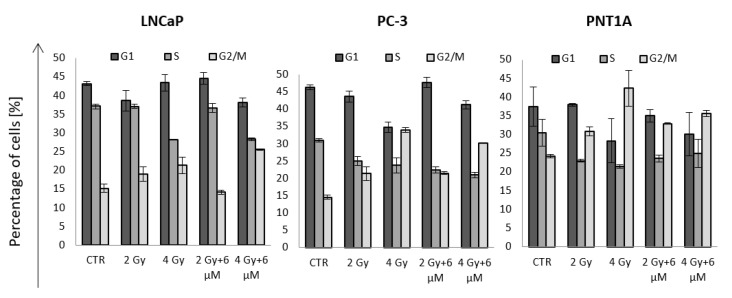
The percentage of cells in the G1, S, and G2 phases of the cell cycle after treatment with nanorods and radiation. The cells were irradiated with 2 and 4 Gy doses following incubation with nanorods at a concentration of 6 μM. LNCaP was irradiated after 6 h of incubation, PC-3 was irradiated after 4 h of incubation, and PNT1A was irradiated after 4 h of incubation. The values presented in the graph are the mean of the obtained results, and the deviation is the standard deviation. The experiment was performed in triplicate.

Lastly, we decided to measure cell cycle distribution 24 h after the irradiation of cells incubated with nanorods (Figure 7). The purpose of the measurement was to analyze the fraction of cells in the G2 phase, which is the radiosensitive phase of the cycle. All three cell lines showed a trend of increasing fraction of cells in G2 with increasing dose of ionizing radiation. Comparing the cells treated with radiation alone and cells additionally incubated with nanorods, we could observe differences in the fraction of cells in G2. In the case of the LNCaP cells, at a dose of 2 Gy, a 4.8% (±0.6%) reduction in G2 phase could be observed, while at 4 Gy, there was an increase of 4.3% (±0.9%) compared to the cells treated with radiation only. The PC-3 cells showed no difference between cells treated with the 2 Gy dose after incubation with GNRs and cells exposed to irradiation alone. In PC-3 cells, there was a 3.8% (±0.2%) decrease in the G2 phase fraction in cells exposed to 4 Gy in combination with GNR compared to the cells irradiated alone. The PNT1A cells showed an increase of 2.9% (±0.2%) in 2 Gy and GNR-treated samples compared to cells treated with 2 Gy alone and a decrease of 6.8% (±0.8%) for cells irradiated with 4 Gy after incubation with GNR compared to cells irradiated with 4 Gy alone.

## 3. Discussion

Radical radiation therapy is an important element of cancer treatment. The data show that 30–50% of patients diagnosed with prostate cancer undergo ionizing radiation therapy [10]. Unfortunately, radiation therapy is non-selective: it damages both cancer and normal cells, which can lead to serious side effects for patients. There is a need for new, improved therapeutic methods that show increased cytotoxicity toward cancer cells and reduced side effects [11]. In the light of the disadvantages of current treatment methods, one of the most important emerging tasks in nanotechnology is to improve the effectiveness of cancer therapy by targeting cancer cells while sparing healthy tissues [12]. Gold nanoparticles turned out to be especially interesting structures due to their complex biological and physical impact on cells. One of the key discoveries made by Matsumura and Maeda [13] in the 1980s revealed that nanoparticles can accumulate in tumors. It led to scientists’ interest in the use of nanoparticles in cancer therapy. In many studies, increased radiosensitivity was observed in GNP-treated cells. However, the majority of studies described this effect in a phenomenological way, i.e., the emphasis was only on the description of the phenomenon itself, without examining its causes. As a result, the mechanisms by which sensitization occurs remain unclear. It is currently postulated that the observed increased radiotoxicity is a result of increased photon induction due to photoelectric effect. The probability of this effect increases with the higher atomic number of the irradiated material and is greater for the radiation delivered at kilovolt energy. Radiation energies obtained from megavolt sources are used in clinical radiotherapy; therefore, this mechanism will not occur. In order to elucidate the biological effects in a more accurate manner, it became necessary to know the significance of the size, concentration, and impact of nanoparticles on the target material such as DNA [14,15]. The main objective of this study was to analyze the capacity of gold nanoparticles to changes in the efficiency of ionizing radiation in human prostate cancer cell lines LNCaP and PC-3 as well as normal prostate cells PNT1A. This model was chosen because radiotherapy is frequently used in the treatment of prostate cancer. We chose two different cancer cell lines to observe if there are differences in biological response, because of their micromolecular characteristics. LNCaP is the androgen-sensitive human prostate adenocarcinoma cell line, whereas PC-3 does not respond to androgens, glucocorticoids, or fibroblast growth factors. In the first stage of the study, the cytotoxicity of gold nanorods (Figure 1) was measured in a wide range of concentrations, i.e., 2–100 μM, at five time points—4, 6, 24, 48, and 72 h (Figure 2). With the increasing GNR concentration, cells showed lower viability. The increased incubation time with nanorods also resulted in higher toxicity. Before being added to the cells, the nanorods were subjected to a triple purification process to ensure the smallest possible amount of CTAB surrounding the nanorods. Nevertheless, we agree that CTAB (surface-bound and dissolved) could have a non-negligible effect on cell response to GNRs. Cells showed close to 50% mean viability after incubation with nanorods in the concentration 30 μM after 24 h, while higher concentrations caused a more pronounced decrease. A similar response between the lines was observed; however, at low concentrations after 72 h of incubation, the normal prostate line showed much lower viability than cancer cells. Woźniak et al. [16] also studied the effect of gold nanorods on cellular response. In their study in the cell lines HeLa and HEK293T, a decrease in metabolic activity at concentrations of 32, 64, and 100 μM was observed. With increasing incubation times, i.e., 24, 48, and 72 h, a constant trend of decreasing viability could be seen. The effect of GNRs was different between the two cell lines. A number of studies [17,18,19] have shown that a general cytotoxic mechanism is initiated during the internalization of GNPs in combination with the inactivation of related enzymes, mitochondrial membrane depolarization, redox cell imbalance, and lysosomal dysfunction in cells. These damages increased the level of reactive oxygen species in cells, inducing apoptosis and accelerating damage to the cell membrane.

The second stage of the work was the analysis of the lateral scattering parameter (SSC) to determine the cellular granularity (Figure 3). It can be assumed that the internalization of nanorods is associated with increased granularity. The highest granularity points seen in the study indicated when the GNR absorption and adsorption reached maximum. Many studies have shown [20,21,22] that the uptake parameter measured by flow cytometry is the most commonly used method to analyze intracellular nanoparticle uptake. Results obtained by Park et al. [23] confirmed that SSC values correlate with the other advanced and recommended method such as inductively coupled plasma-mass spectrometry (ICP-MS). However, the change in SSC parameter can also be related to the nanorods adsorbed on the surface. The observed uptake values may also depend on the shape of the nanoparticles used in the experiment. A series of low concentrations were used, i.e., 2, 4, 6, 8, and 10 μM and incubation times of 2, 4, 6, and 8 h. As a result, a significant change in granularity depending on the incubation time and the GNR concentration was detected. In the LNCaP cell line, an increase in granulation has been observed from the 4th hour of incubation with a maximum value for 6 h and a further decrease for concentrations above 6 μM. In PC-3 cells, an upward trend in SSC was observed after an incubation time of 4 h followed by a gradual decrease. The PNT1A cell line was characterized by a very mild increase in SSC for concentrations below 6 μM over a time up to 4 h. GNR at higher concentrations caused a significant increase in SSC, while at subsequent time points, a strong decrease in granularity could be observed. Chithrani et al. [24] investigated the dependence of uptake on the shape of nanoparticles. They observed that spherical gold nanoparticles with a core diameter of 74 nm and 14 nm were well captured by the cells, but the maximum uptake occurred for nanoparticle size of 50 nm. Furthermore, they checked that shorter nanorods were better absorbed by cells than longer GNRs. It had been shown that the ability of cancer cells to take up spherical forms of GNP is much higher than rod-shaped nanoparticles [25].

The next part of the work was cell cycle analysis (Figure 4). Cells were incubated with nanorods to determine cell cycle distribution at maximum internalization of the nanorods. The LNCaP cell line showed a small increase in cell accumulation in the G2 phase, which is the phase sensitive to ionizing radiation. PC-3 and PNT1A cells did not show any difference in the cell cycle. It suggests that gold nanorods do not determine the radiosensitivity effect by blocking cells in the G2 phase, which was one of the main assumptions to be verified. Roa et al. [9] analyzed the effect of spherical, functionalized gold nanoparticles on DU-145 prostate cancer cells and human MRC5 fibroblasts. After 2 h treatment of the DU-145 cells with nanoparticles at a concentration of 15 nM, the fraction of cells in the G2 phase increased by 11% compared to the control group. Then, cells were irradiated. As a result, a lower fraction of surviving cells was observed, proving the possibility of regulating the cycle with nanoparticles. In response to radiation, cells activate cell cycle checkpoints in G1, S, and G2 phases to repair genomic defects, maintain their integrity, or prevent cell division by cell death mechanisms activation [26]. On the basis of literature reports [9], it was assumed that nanoparticles may potentiate the accumulation of cells in the G2 phase after exposure. In our experiments, no differences were observed in the fraction of cells in a given phase of the radiation-treated cells previously incubated with nanorods in comparison with the cells exposed to radiation only. This effect is different than in the work cited above. 

In addition, the level of reactive oxygen species induced in cells by incubation with gold nanorods was measured (Figure 5), as several sources show the effect of nanoparticles on ROS level [27,28]. After incubating cells with gold nanorods, small differences in ROS induction were observed. However, a dominant trend can be seen for concentrations above 6 μM: the level of reactive oxygen species increases with increasing GNR concentration. It is possible that lower levels of ROS induction are caused by the relatively large size of nanorods used in this study. Studies have shown that the reduction of GNP size correlates with increased potential for deeper penetration, more effective internalization by cells, and increased toxicity. It has been shown that GNPs exert cytotoxicity by inducing oxidative stress. Johnston et al. [29] investigated the effect of 1.4 nm GNPs on HeLa cervical cancer cells. Analyses showed an increased production of reactive oxygen species and higher oxidative stress, which led to protein and lipid oxidation, severely impaired mitochondrial function, and ultimately cell death. Mitochondria seem to play a key role in the cytotoxicity response to reactive oxygen species because the data indicate a loss of function caused by high levels of intracellular ROS. It is likely that the mitochondrial membrane oxidizes, which disrupts its potential and causes more superoxide anions to enter the cytosol, which in turn can be transformed into H2O2 molecules that further diffuse through the membranes and damage DNA [30].

Lastly, we analyzed the surviving fraction (SF) after incubation with GNR followed by irradiation (Figure 6 and Figure 7). For this purpose, one concentration of 6 μM was chosen because of the high granularity value and relatively low toxicity. The PC-3 and PNT1A cells were irradiated with 2 and 4 Gy doses after 4 h of incubation with nanoparticles, and for the LNCaP cells, the incubation time was 6 h. Analyzing SF values for the LNCaP line, there was no statistically significant difference between cells incubated with nanorods, which are treated with a dose of 2 Gy and 4 Gy, and cells irradiated alone. A different relationship was observed for the PC-3 line. A statistically significant difference was noted for the 2 Gy dose. The PNT1A cells did not show a statistically significant difference in survival after incubation with GNRs and irradiation with 2 Gy and 4 Gy doses compared to the cells that were irradiated without GNR incubation. Several research groups have focused on the effect of cell hypersensitivity to ionizing radiation. Chithrani et al. [6] studied the effect of GNP size, concentration, and radiation energy on Hela cells. Studies had shown that 50 nm nanoparticles are internalized in Hela more efficiently than 14 nm or 74 nm GNRs. Using 220 kVp x-rays, the highest sensitivity increase was observed for 50 nm GNP. It was found that the level of radiation sensitivity depends on the concentration of GNPs and correlates with the number of intracellular nanoparticles but not with a total concentration of intracellular gold. In other studies, Rahman et al. [31] tested gold nanoparticles on bovine aortic endothelial cells. They reported that the dose enhancement factor while using nanoparticles at a concentration of 0.5 mM and a diameter of 1.9 nm at 6-MeV and 12-MeV electron energy is 2.9 and 3.7, respectively. These studies, together with the growing evidence of GNP’s biological activity, suggest that the mechanism of radiosensitization may be due to other effects than just increasing the dose by using a high atomic number material. Jain et al. [32] studied the cellular uptake, intracellular location, and cytotoxicity of nanoparticles in normal L132 cells, DU145 prostate cancer, and MDA-MB-231 breast cancer cells. GNP uptake occurred in all cell lines and was highest in MDA-MB-231 cells with nanoparticles accumulating in cytoplasmic lysosomes. In MDA-MB-231 cells, sensitizer enhancement ratios (SERs) of 1.41, 1.29, and 1.16 were observed using X-ray energy of 160 kVp, 6 MV, and 15 MV, respectively. No significant effect was observed in L132 or DU145 cells at kV or MV energies (SER 0.97–1.08). Exposure to nanoparticles did not increase the radiation-induced double-strand breaks of the DNA nor did it inhibit repair mechanisms. The authors suggest [14] that GNP-sensitizing properties could probably be strongly dependent on the nature of their coating. However, the divergence from the radiation-sensitive GNP effects can also be attributed to the different dimensions of the GNPs used as well as the type of tested cancer cells. Another study was conducted on the effect of 16 nm gold nanoparticles on human erytroleukemia K562 cells after gamma irradiation. Minimal cytotoxicity (<10%) has been demonstrated at low concentrations of gold nanoparticles (<100 μg/mL), which increased significantly to 58% when using the highest concentration 600 μg/mL of gold nanoparticles, showing dose-dependence [33]. 

## 4. Materials and Methods

### 4.1. Gold Nanorods Synthesis

Synthesis of gold nanorods was performed according to the seed-mediated technique published by Nikoobakht and El-Sayed [34]. Seeds were prepared by continuous mixing 5 mL of 0.2 M cetyl trimethylammonium bromide (CTAB) solution with 5 mL of 0.5 mM of tetrachloroauric acid (HAuCl_4_) solution and then the addition of 0.6 mL of 0.01 M ice-cold NaBH_4_ solution while vigorously stirring. This process resulted in the rapid change of the color of the solution from yellow to brown, signaling a process of seeds creation. Afterwards, the seed solution was kept undisturbed in 25 °C. In the second probe, 2.5 mL of 0.2 M CTAB solution was gently stirred in temperature of 28 °C. During stirring, 0.075 mL of 4 mM of AgNO_3_ solution and 2.5 mL of 0.001 M of HAuCl4 solution were added. Furthermore, 35 μL of 0.0788 M L-ascorbic acid as a reducing agent was added. The solution became colorless. Finally, when the temperature of the solution again reached 28 °C, 6 μL of the previously prepared seed solution was added. The solution was vigorously stirred, and after 10–20 min, the color gradually changed to violet-blue, indicating the formation of nanorods. The solution was kept at 28 °C until the moment of purification. To discard excess CTAB, the final reaction product was purified by centrifugation (13,000 rpm, 4 × 20 min, 30 °C), and the supernatant solution, containing surfactant. This purification procedure was repeated three times. 

### 4.2. Transmission Electron Microscopy (TEM)

First, 5–10 μL of aqueous dispersion was placed on a copper grid covered with a formvar–carbon membrane (300 mesh, Ted Pella Inc., Redding, CA, USA) and dried at room temperature. HRTEM images were using Jeol ARM 200F TEM microscope operating at 200 kV and images were analyzed using ImageJ. Over 100 nanorods were measured for analysis.

### 4.3. Cell Culture

The LNCaP, PC-3, and PNT1A cell lines used in the experiments were maintained under standard conditions, at 37 °C, in an atmosphere enriched with 5% CO_2_, saturated with water vapor incubator (Binder, Tuttlingen, Germany). The basic culture media were Roswell Park Memorial Institute (RPMI) medium 1640 (for LNCaP and PNT1A cells) (Biowest, Nauille, France) and Dulbocco’s Modified Eagle’s Medium (DMEM) (in case of PC-3 lines) (Biowest, Nauille, France) supplemented with 10% fetal bovine serum (FBS) (Biowest, Nauille, France) with the addition of antibiotic agents (penicillin/streptomycin at a final concentration of 1%) (Merck Millipore Corporation, Darmstadt, Germany). Cell culture reaching min. 80% confluence was passaged every 2–3 days. The experiments were carried out in sterile conditions, under a biosafety cabinet with laminar air flow (Telstar, Madrid, Spain).

### 4.4. Estimation of GNR Concentration

First, 0.5 mL of the prepared nanorods solution was added to five eppendorfs. After complete sedimentation of the nanorods to the bottom, the excess amount of supernatant with distilled water was removed and allowed to evaporate by itself. After 48 h, the remaining dried content was weighed. On the basis of the obtained values, the concentration of nanorods in the solution was assessed at 0.3917 g/L. The concentration was converted to the other unit—µM by a calculation formula using the mole mass and volume of gold nanorods. Concentrations used in experiments—2, 4, 6, 10, 30, 60 and 100 μM (0.0004; 0.0008; 0.0012; 0.0020; 0.0060; 0.0120; 0.0200 g/L)

### 4.5. MTT Assay

Cells were plated at a predetermined concentration onto 96-well flat-bottomed plates (LNCaP—10000 cells/well, PC-3—5000 cells/well, PNT1A—7500 cells/well). After 24 h, nanorods (initial concentration 1150 µM) were added at final concentrations of 2, 4, 6, 10, 30, 60, and 100 μM at final volumes of 200 µL per well. The tests were closed after 4, 6, 24, 48, and 72 h of incubation of cells with nanorods in the culture medium. Then the medium was removed and the new medium containing MTT (3-(4,5-dimethylthiazol-2-yl)-2,5-diphenyltetrazolium bromide) (Affymetrix, Cleveland, OH, USA) at a final concentration of 0.5 mg/mL was added to the cell culture. Cells were incubated for 2 to 4 h in an incubator depending on the metabolic rate for a given cell line. Then, the medium was discarded, and 100 μL DMSO (Thermo Scientific, Waltham, MA, USA) was added per well to dissolve the formazan crystals formed. The results were read with a Multiskan plate reader at 570/590 nm, background 655 nm (Thermo Scientific, Waltham, MA, USA).

### 4.6. Nanorods Interaction

The interaction of gold nanorods with cells (uptake and surface adsorption) was measured according to the technique published by Park et al. [23]. Cells were plated at 300,000 cells/well in a 6-well plate. After 24 h, the medium was removed, and then, a solution of the medium and nanorods at the final concentrations of 2, 4, 6, 8, and 10 μM was added. Cells were incubated for 2, 4, 6, and 8 h. To discard excess GNR from the sample, cells were collected, purified by centrifugation, and suspended in Phosphate Buffered Saline (PBS) (Biowest, Nuaille, France) for cytometric analysis, using a Cytoflex Beckmann Coulter cytometer (Beckman Coulter Life Sciences, Indianapolis, IN, USA). Fluorescence was measured at 611 nm by analyzing the side scatter parameter (SSC). Analysis of the obtained results was performed using FlowJo v10.

### 4.7. Cell Cycle

A total of 300,000 cells per well were seeded in a 6-well plate. After 24 h, the medium was removed, and a solution of medium and nanorods was added at the final concentrations of 2, 4, 6, 8, and 10 μM in a volume of 2 mL per well. After 4, 6, and 24 h, cells were collected and fixed in 70% ethanol solution (POCh, 44101 Gliwice, Poland). The prepared samples were stored at −20 °C for further analysis. The pellet was suspended in 200 μL of a mixture consisting of 10 μL of 1 mg/mL propidium iodide (Cayman Chemical Company, Ann Arbor, MI, USA), 2 μL of 10 mg/mL RNase I enzyme (PanReac AppliChem, ITW Reagents, Chicago, IL, USA), and 188 μL of PBS solution. Samples were incubated for 30 min at 37 °C, protected from light. Fluorescence was measured at 611 nm. The obtained results were analyzed using FlowJo v10.

### 4.8. ROS Assay

Cells were seeded at 200,000 cells/well. A 1:1000 solution of 2’,7’-Dichlorofluorescin Diacetate DCFH-DA (Merck Millipore Corporation, Darmstadt, Germany) in the culture medium without fetal bovine serum as prepared. After removal of the medium, dye was applied to the cells, which were then incubated for 45 min in an incubator at 37 °C. After this time, the dye was washed off with PBS. A solution of complete culture medium with gold nanorods at 2, 4, 6, 8, and 10 μM in a volume of 2 mL per well was added to the cells, followed by incubation for 4 h (PC-3, PNT1A) or 6 h (LNCaP). Cells were collected, suspended in PBS, and analyzed by flow cytometry. The measurement was performed using a flow cytometer on the fluorescein isothiocyanate-height (FITC-H) channel (at 485 nm excitation wavelength and 527 nm emission).

### 4.9. Clonogenic Assay

The irradiation was carried out using a Gamma Cell^®^ 1000 Elite device (BestTheratronics Ltd., Vancouver, BC, Canada) with a dose rate of 2.5 Gy/min using a closed source Cs-137 with an activity of 20.4 TBq. Cells in suspension were exposed to 2 and 4 Gy doses. After irradiation, the cells were plated on 6-well plates followed by incubation for specified time (LNCaP: 6 days, PC-3: 12 days, PNT1A: 14 days). Clonogenic assays were closed when control colonies contained a minimum of 50 cells. The medium was removed, the plate was washed with 2 mL PBS, and fixed with 2 mL of denatured ethanol. After removing the alcohol, the plates were stained with approximately 2 mL of Coomassie Blue solution (Merck Millipore Corporation, Darmstadt, Germany) and incubated for approximately 20 min. Then, the buffer was removed and the plates were washed in warm water and dried. Clonogenic tests were photographed using the ChemiDoc Touch Bio-Rad system (Hercules, CA, USA). Automatic colony counting was performed using the Gene Tools Syngene program. After counting the colonies, plating efficiency (PE) was calculated expressing the ratio of counted colonies to seeded cells, i.e., PE = number of counted colonies/number of seeded cells. Then, the surviving fraction (SF) was calculated as the ratio of PE irradiated cells to PE control cells.

### 4.10. Statistical Analyzes

Statistical analysis of the results was carried out with the Statistica V12.5 (purchased from: StatSoft Polska Sp. z o. o., Kraków, Poland) and Microsoft Excel (purchased from: Microsoft Sp. z o. o., Warszawa, Poland). To analyze the normal distribution of the studied groups, the Shapiro–Wilk test was used. The analysis of the relationship between the two groups was made using the Student’s *t*-test for two groups. The results of *p* level ≤ 0.05 was assumed as statistically significant.

## 5. Conclusions

To conclude, gold nanorods showed toxic effects on all cell lines, both normal and cancerous. Low GNR concentrations did not significantly reduce proliferation. Analysis of the lateral scattering parameter showed differences in cellular granularity, which indicates that nanorods are absorbed by cells. The highest granularity value reflected the maximum internalization of nanorods into the intracellular area after 4 and 6 h of incubation with GNR. The lack of significant changes in the cell cycle at the time point of maximum uptake of nanorods may indicate insufficiently long incubation time of cells with GNR to observe biological effects. Nanorods induced the production of reactive oxygen species, which is one of the factors determining the effect of cytotoxicity. Survival analysis of the cells (SF) irradiated after incubation with GNRs and cells irradiated alone showed no statistically significant differences in the case of LNCaP and PNT1A lines. However, statistically significant decrease of SF was observed in PC-3 cells treated with irradiation and GNRs compared to the cells irradiated alone. Presumably, the effect induced by nanorods is closely related to the occurrence of biological effects depending on the characteristics of a given cell line. In the normal prostate cell line, no changes in cell proliferation were observed, which may indicate some selectivity for cancer cells.
